# Digital morphological data can generate accurate pre-emergence herbicide dose-response curves in *Chenopodium album* L

**DOI:** 10.3389/fpls.2026.1779398

**Published:** 2026-04-21

**Authors:** Olivia Landau, Jesus Lopez Borges, Raissa Na-ah, Luigi Peracchi, Franco Sanchez-Izurieta

**Affiliations:** 1 United States Department of Agriculture, Agricultural Research Service, Wheat Health, Genetics, and Quality Research Unit, Pullman, WA, United States; 2 Department of Crop and Soil Sciences, Washington State University, Pullman, WA, United States

**Keywords:** atrazine, digital biomass, digital phenotyping, dose-response, fomesafen, morphological data, spectral data

## Abstract

**Introduction:**

Herbicide dose-response assays are routinely implemented to compare herbicide resistance among weed biotypes, which requires plant biomass to estimate the dose that reduces growth by 50% relative to untreated plants (GR_50_). The Phenospex TraitFinder is a high-throughput, non-destructive, digital phenotyping system that collects data from 7 spectral parameters and 13 morphological parameters, including Digital Biomass (DB), which offers the opportunity for researchers to eliminate the time and labor associated with manual biomass collection. However, DB is the product of 3D Leaf Area and Plant Height (PH) Mean, making it a measurement of plant volume and an indirect indicator of biomass. While DB is highly correlated with true biomass, digitally collected plant volume data has not been implemented for dose-response assays or assessed for accuracy relative to true biomass data. Additionally, inaccurate PH measurements could impact the accuracy of DB measurements.

**Methods:**

This study sought to assess the accuracy and utility of DB and the 19 remaining parameters in dose-response assays by comparing dose-response curves and GR_50_ estimates generated from digital data and fresh biomass (FB) data. Accuracy of PH measurements were also assessed by comparing digital and manual measurements with the paired t-test. Pre-emergence dose-response assays using fomesafen and atrazine were implemented with common lambsquarters (*Chenopodium album* L.). At 21 days after treatment, manual measurements of FB and PH were collected following digital data collection.

**Results:**

Consistently strong correlations (*r* = 0.97, *P* < 0.05) were observed between digitally collected data and their equivalent manual measurements. Comparisons of the dose-response curves indicated that only 3D Leaf Area, DB, Convex Hull Area, Projected Leaf Area, and Voxel Volume Total generated highly similar curves and GR_50_ estimates relative to FB data, indicating that any one or all of these parameters could be utilized instead of FB. Small differences (approximately 1.06 to 1.77 mm) between manual and digital PH measurements were identified with the paired t-test, but since DB consistently produced similar dose-response curves and GR_50_ estimates relative to FB, these differences did not impact the accuracy of DB measurements.

**Discussion:**

Without requiring manual biomass collection, turnaround time for dose-response and other phenotyping assays decreases and allows faster sharing of research. Furthermore, herbicide-resistant plants can be preserved for phenotyping at later growth stages, tissue collection, and to produce progeny for future experiments.

## 1 Introduction

After herbicide-resistant weeds have been identified under field conditions, seeds from these biotypes are typically sent to weed scientists for further evaluation. Dose-response assays are a staple within weed science for quantitatively characterizing herbicide-resistant weed biotypes, and they have been utilized for numerous herbicides and weed species (Brunharo and Hanson, 2018; Strom et al., 2019; Lu et al., 2020; Rodriguez et al., 2021; Ribeiro et al., 2023; Randell-Singleton et al., 2024; Vijaya Bhaskar et al., 2025; Xu et al., 2025). These assays are conducted by subjecting plants to a series of increasing herbicide doses that capture the herbicide injury spectrum, and then biomass data is plotted as a function of increasing herbicide doses. Generally, this data will generate a negative, sigmoidal curve and allow for growth reduction 50 (GR_50_) estimations using a log-logistic model (Seefeldt et al., 1995; Knezevic et al., 2007; Keshtkar et al., 2021). The GR_50_ is the dose needed to cause a 50% reduction in plant biomass relative to an untreated control (Seefeldt et al., 1995). These estimates are typically compared by calculating the resistance index, which is the quotient of the suspected-resistant population GR_50_ and the sensitive population GR_50_ (Burgos et al., 2013; Keshtkar et al., 2021). If the GR_50_ estimate in the suspected-resistant population is greater than the sensitive population, then these data indicate the population is herbicide-resistant, and further investigation of the resistance mechanism is conducted (Vijaya Bhaskar et al., 2025; Xu et al., 2025).

The primary challenges of these experiments are the time and labor required to collect biomass, which is partly due to the inherent large size of these experiments. In addition to an untreated control, several herbicide doses are needed to capture the injury spectrum and allow for accurate estimation of the GR_50_ (Burgos et al., 2013). At a minimum, these experiments include two populations, a sensitive and a suspected-resistant population, and each dose would be applied to at least three biological replicates per population. These experiments are repeated at least two to three times to demonstrate repeatable results and to increase the total number of biological replicates to approximately 10 (Burgos et al., 2013). Thus, if there is a need to add more biological replicates, doses, or populations to the experiment, the required labor rapidly increases. Researchers commonly examine multiple resistant populations (Ma et al., 2016; Obenland et al., 2019; Shrestha et al., 2019; Rangani et al., 2021; Priess et al., 2022; Yanniccari et al., 2023), and there is an increasing trend to utilize multiple sensitive populations (Strom et al., 2019; Priess et al., 2022; Shyam et al., 2022; Borgato et al., 2024). Given these labor requirements, it is logical for researchers to investigate methods to mitigate these challenges and increase their capacity.

Multispectral imaging has been an effective, high-throughput, non-destructive alternative to traditional plant phenotyping of herbicide injury, especially for field experiments (Huang et al., 2015; Da Silva et al., 2019; Xia et al., 2022). Recently, researchers have demonstrated the potential utility of multispectral imaging for use in leaf disk dose-response assays by using this methodology to distinguish between putative resistant and sensitive plants subjected to glufosinate and fomesafen (Jones et al., 2023a, Jones et al., 2023b). While these results are intriguing and indicate the potential for rapid detection of glufosinate- or fomesafen-resistant biotypes, the need for whole-plant phenotyping of herbicide injury will remain, especially when characterizing novel herbicide resistance mechanisms. A high-throughput, non-destructive option that allows for the use of whole-plant dose-response assays is the Phenospex TraitFinder (Phenospex, Netherlands), which is a digital phenotyping workstation equipped with an adjustable bench and two PlantEye F600 3D scanners. As the scanners move across the bench, they each shine a laser light and utilize the laser line triangulation principle to build a 3D model of the scanned plants (Phenospex, 2024). When plants are scanned, data for 13 morphological parameters and 7 spectral parameters are collected. These parameters are classified as either digital or technical (**Table 1**): both classifications highly correlate with one or more plant traits, but the degree of correlation varies across species for technical parameters, while the degree of correlation for digital parameters is more consistent across species (Phenospex, 2024). All 20 parameters have potential utility in dose-response assays. However, digital biomass (DB) is of great interest as a replacement for destructive biomass collection, but it is calculated as the product of 3D Leaf Area (3DLA) and Plant Height (PH) Mean (**Table 1**), resulting in DB being a measurement of plant volume and an indirect indicator of biomass.

**Table 1:** Information regarding Phenospex TraitFinder PlantEyes and parameters.†

PlantEye color channel and corresponding wavelength				
Color channel	Abbreviation	Peak wavelength (nm)		
Red	R	624-634		
Green	G	530-540		
Blue	B	465-485		
Near-infrared	NIR	720-750		
Morphological Phenospex TraitFinder parameter information				
Parameter name	Abbreviation	Formula/Description	Value Range	Type^‡^
3D leaf area (mm^2^)	3DLA	The sum of all calculated triangle surfaces within plant structure.	0 - ∞	Digital
Canopy light penetration depth (mm)	CLPD	The depth at which laser light can penetrate the plant’s canopy.	0 - ∞	Digital
Convex hull area (mm^2^)	CHA	The maximum space a plant could occupy at its current size.	–	Technical
Convex hull area coverage (%)	CHAC	The percentage of CHA occupied by the plant.	–	Technical
Convex hull circumference (mm)	CHC	The distance around the convex hull.	–	Technical
Convex hull max width (mm)	CHMW	The distance of the longest straight line that can be drawn on the CHC.	–	Technical
Convex hull aspect ratio (%)	CHAR	The ratio between CHMW and the perpendicular line that intersects CHMW line at its midpoint. It correlates with the growth stage of a plant and can potentially distinguish between different morphologies.	–	Technical
Plant height average (mm)	PH Mean	PH Mean is measured from the base of the plant to the mean of the heights within the top 10% of the plant. The measurement prioritizes stability over accuracy by minimizing the effect of small movements caused by wind, external factors, or diurnal plant rhythms. It correlates highly with overall plant height.	0 - ∞	Digital
Plant height max (mm)	PH Max	PH Max is measured from the base of the plant to the absolute highest point of the plant. It correlates highly with overall plant height.	0 - ∞	Digital
Projected leaf area (mm^2^)	PLA	PLA is calculated by mapping all triangles onto an X-Y plane and is the total area of a plant as seen from a overhead perspective.	0 - sector size	Digital
Digital biomass (mm^3^)	DB	The product of 3DLA and PH Mean.	0 - ∞	Digital
Surface angle average (°)	SA Mean	The mean angle of the triangles relative to the height axis. It correlates with leaf angle.	0 - 90	Technical
Voxel volume total (mm^3^)	VVT	Similar to how 2D images are made up of pixels, 3D images are made up of voxels. VVT is the total number of visible voxels multiplied by the voxel volume.	–	Technical
Spectral Phenospex TraitFinder parameter information^§^				
Parameter name	Abbreviation	Formula/Description	Value Range	Type
Normalized difference vegetation index mean	NDVI Mean	$NDVI=\;\frac{\left(NIR-R\right)}{\left(NIR+R\right)}$	-1 - 1	Digital
Green leaf index mean	GLI Mean	$GLI=\;\frac{\left(2\times G-R-B\right)}{\left(2\times G+R+B\right)}$	-1 - 1	Digital
Plant senescence reflectance index mean	PSRI Mean	$PSRI=\;\frac{\left(R-B\right)}{\left(NIR\right)}$	-1 - 1	Digital
Normalized pigment chlorophyll index mean	NPCI Mean	$NPCI=\;\frac{\left(R-B\right)}{\left(R+B\right)}$	-1 - 1	Digital
Hue (°) mean	H Mean	H represents an angle on the color wheel. Red is 0° and 360°, green is 120°, and blue is 240°.	0 - 360	Technical
Lightness (%) mean	L Mean	L indicates the brightness of color; 0 is dark and 100 is bright.	0 - 100	Technical
Saturation (%) mean	Sat Mean	Sat represents the intensity of color; 0 is unsaturated and 100 is saturated.	0 - 100	Technical

^†^The following information regarding the PlantEyes and parameters can be found in the HortControl user manual. (Phenospex, 2024)

^‡^Digital parameters highly correlate with plant traits regardless of plant species. Technical parameters also correlate closely with plant traits, but the degree of correlation varies across plant species.

^§^The means for the following parameters are calculated across all the 3D points of the plant model.

Currently, there are several examples of Phenospex technology being utilized for phenotyping of morphological and spectral parameters, but the primary focus has been on crops rather than weed species. Some examples include soybean (*Glycine max*), mungbean (*Vigna radiata*), common bean (*Phaseolus vulgaris*), cowpea (*Vigna unguiculata*), lima bean (*Phaseolus lunatus*), okra (*Abelmoschus esculentus*), wheat (*Triticum aestivum*), tomato (*Solanum lycopersicum*), French marigolds (*Tagetes patula*), and grass sod (Manavalan et al., 2021; Schafleitner et al., 2021; Miroshnichenko et al., 2022; Bazhenov et al., 2023; Quijia Pillajo et al., 2023, Quijia Pillajo et al., 2024; Zieschank and Junker, 2023; Galba et al., 2025; Mohammed et al., 2025; Quijia-Pillajo and Jones, 2025). While these examples demonstrate the utility of this technology, researchers commonly only reported results for a few parameters, and comparisons of the digital data to manually collected data were rarely investigated. The 3DLA, DB and PH parameters are the most commonly reported morphological parameters (Manavalan et al., 2021; Schafleitner et al., 2021; Miroshnichenko et al., 2022; Bazhenov et al., 2023; Quijia Pillajo et al., 2023, Quijia Pillajo et al., 2024; Mohammed et al., 2025; Quijia-Pillajo and Jones, 2025), while normalized difference vegetation index was the most commonly reported spectral parameter (Schafleitner et al., 2021; Miroshnichenko et al., 2022; Bazhenov et al., 2023; Quijia Pillajo et al., 2023, Quijia Pillajo et al., 2024). The few existing examples comparing digital and manually collected data involved correlating DB to dry biomass in durum wheat (*Triticum durum*) and rye (*Secale cereale*) (Bazhenov et al., 2023), correlating manually and digitally collected PH measurements in *Petunia × hybrida* (Quijia Pillajo et al., 2023), and correlating both manually and digitally collected biomass and PH in hemp (*Cannabis sativa*) (Singh et al., 2024).

There are some examples of this technology being utilized for phenotyping stress treatments, including flooding stress in okra (Schafleitner et al., 2021) and heat stress in tomato (Mohammed et al., 2025). However, when it comes to herbicide-treated plants, the Phenospex TraitFinder has had very limited utilization and has not been implemented for dose-response assays. To date, the only example with herbicide-treated plants involved evaluating the efficacy of different clopyralid formulations with and without a new surfactant on cocklebur (*Xanthium strumarium*) by comparing DB, PH, 3DLA, and spectral data over time (Mirgorodskaya et al., 2023). Again, digital data was not compared to manually collected data.

While it is reasonable for researchers in the previous phenotyping experiments to assume their results and conclusions would have been the same regardless of whether they utilized DB or conventional biomass, it is worthwhile to investigate the accuracy of DB for dose-response assays since inaccurate measurements could artificially inflate or deflate GR_50_ estimates. We hypothesize that if DB accurately reflects fresh biomass (FB), then DB data should produce similar dose-response curves and GR_50_ estimates compared to FB data. This hypothesis also extends to all data collected by this system. Additionally, the system collects two PH measurements, PH Max and PH Mean, and the PH Mean parameter is especially relevant to compare to manual measurements of PH (PH True) due to it being an essential component for calculating DB (**Table 1**). Thus, we hypothesize that inaccurate measurements of PH Mean will have a commensurate effect on DB measurements.

Given that the Phenospex Traitfinder is advertised to measure plants and plant organs down to a size of 0.5 mm (Phenospex, 2021), pre-emergence herbicides were chosen for the following dose-response assays since the surviving plants treated with relatively high herbicide doses can commonly be a few millimeters in size, making this scenario ideal for testing the lower limits of detection. The objectives of this research are (i) compare manual measurements of FB and PH to the equivalent digitally collected parameters through Pearson correlation analysis and the paired t-test, and (ii) compare dose-response curves and GR_50_ estimates between FB data and digital data to determine GR_50_ estimation accuracy and utility in dose-response assays.

## 2 Materials and methods

### 2.1 Plant material, chemicals, herbicide application, and growth conditions

Seeds of common lambsquarters (*Chenopodium album* L.) were collected in September 2018 at the Palouse Conservation Field Station (46°45’N 117°11’W) and stored at room temperature until these experiments were initiated. Fomesafen (Reflex^®^, Syngenta, Greensboro, NC) and atrazine (Aatrex 4L^®^, Syngenta, Greensboro, NC) were selected because they are two pre-emergence herbicides labelled for control of common lambsquarters (Anonymous, 2022, Anonymous, 2024). Given that these herbicides were rarely implemented at the collection site, this population is likely sensitive to fomesafen and atrazine. Fomesafen inhibits protoporphyrinogen oxidase, which is an enzyme required for chlorophyll biosynthesis, and atrazine inhibits photosynthesis by binding to the D1 subunit of photosystem II (Székács, 2021). Both herbicides induce the production of reactive oxygen species and injury symptoms of chlorosis and necrosis (Traxler et al., 2023). However, the visual appearance of injury is distinct; fomesafen rapidly causes injury wherever the plants come into contact with the herbicide, and atrazine displays symptoms in the interveinal areas and margins of the leaf tissue before progressing into necrosis of the whole leaf (Anderson and Hartzler, 2020). To increase germination, dry seeds were stored at 4 °C for seven days. Seeds were pre-germinated on water-soaked filter paper in growth chamber (Conviron Gen1000, Controlled Environments, Winnipeg, Canada) conditions for five days under a 28/22 °C day/night and 16/8-h photoperiod. The LED lights in the growth chamber provided 100 μmol m^−2^s^−1^ photon flux. Only common lambsquarters seeds with approximately 1–3 mm of radicle growth were utilized for sowing. Seeds were planted at a depth of 5 mm in 281 ml pots containing moist soil, and one seed was planted per pot. The soil consisted of 2:1:1 mixture of field soil, peat, and sand (pH 6.0 and 2.5% organic matter). Field soil was a Shano silt loam (coarse-silty, mixed, superactive, mesic Xeric Haplocambids) collected at the Washington State University Lind Dryland Research Station (47°00’N 118°34’W).

Herbicides were applied using a compressed-air research sprayer (Generation III Research Sprayer, DeVries Manufacturing, Hollandale, MN) fitted with a TeeJet^®^ SS8001E nozzle set to 46 cm above the soil surface and calibrated to deliver 140 L ha^−1^ at 207 kPa. The seven doses for atrazine ranged from 0.28 to 280 g ai ha^-1^, and the seven doses for fomesafen ranged from 0.14 to 140 g ai ha^-1^. All doses were spaced by a factor of 3.16. For context, the lowest recommended doses for control of common lambsquarters are 560 and 280 g ai ha^-1^, respectively (Anonymous, 2022, Anonymous, 2024). Eighteen pots were treated with each dose, and each pot represents one biological replicate. Water was applied to 18 untreated control pots. All pots were moved to a greenhouse with a 16/8-hour photoperiod, a daytime deadband of 21/23°C, and the nighttime deadband of 13/15°C. Natural light was supplemented with halogen lamps that provided 1700 μmol m^−2^s^−1^ photon flux. Pots were arranged in a randomized complete block design where each block contained one biological replicate from each dose. The soil moisture was maintained with an overhead misting system that delivered approximately 14 ml water daily. Experiments for each herbicide were conducted twice, resulting in each dose having 36 biological replicates.

### 2.2 Data collection

At 21 days after treatment (DAT), plants were scanned with the Phenospex TraitFinder (**Supplementary Figure S1**). The 21 DAT timepoint was chosen because it is a commonly utilized timepoint for pre-emergence dose-response assays (Ma et al., 2016; Schwartz-Lazaro et al., 2017; Strom et al., 2019) and provided a wide range of differentially sized plants for data collection (**Supplementary Figures S2**, **S3**). Experimental configuration was completed within HortControl, the operating system for data management, which required inputs, such as pot height, cut-off height, and color filter parameters (**Supplementary Figure S2**). HortControl also requires block layout configuration, which designates the location of plants for data collection. In general, the block layout is a grid where each cell of the grid contains one plant from which data is collected. For current experiments, the block layout was a 3x6 grid, where each cell had dimensions of 115 x 103 mm (**Supplementary Figure S2**). To ensure detection of small plants by the PlantEye F600 3D scanners, pots were elevated by placing them upon two RL200 trays (24” L x 12” W x 6.75” H), which brought the pot height up to 395 mm and was noted in the HortControl block layout configuration (**Supplementary Figures S1**, **S2**). The training-based color filter was implemented across all scans to ensure accurate discernment between plant material and non-plant material, especially for plants that were only a few millimeters in height (**Supplementary Figure S3**).

As mentioned previously, the two PlantEye scanners generate 3D models of plants through the laser line triangulation principle, which builds a model of the plants based on the disruptions in the laser line (Phenospex, 2024). During the scanning process, the two scanners shine and simultaneously collect reflectance data for the laser, Red, Green, Blue, and Near-Infrared light (**Table 1**). Each data point in the resulting 3D model contains not only coordinate information but also spectral data. These scanners move at a speed of 75 mm s^−1^, and each scanner captures images at a frame rate of 166.6 frames s^−1^. The x, y, and z resolutions are 0.304, 0.45, and 0.065 mm, respectively (Phenospex, 2025b, Phenospex, 2025a). Within HortControl raw data from the PlantEye scanners is subjected to the data processing chain, which is called Phena 2.0. The steps occur in the following order: Transform, Segment, Triangulate, Split, Merge, and Calculate (Phenospex, 2024). In summary, raw data is transformed to correct the coordinates from the scanner’s perspective to the user’s perspective, groups of data points are segmented within the 3D point cloud using an algorithm based on region growing techniques, sets of at least 3 data points are merged to form triangles, the image is split along the x and y axis to form equal sized rectangles, the images acquired from each PlantEye are merged to remove repetitive information, and data for the 20 plant parameters are calculated (Phenospex, 2024). Details on plant parameter calculations are provided in **Table 1**.

After scanning was completed, plants were cut at the soil line, and manual measurements of PH True and FB were recorded. PH True was measured from the base of the stem to the highest point of the vegetative tissue. FB was collected by cutting plants at the soil line and immediately weighing the plants with a Secura semi-micro balance (Sartorius, Goettingen, Germany).

### 2.3 Statistical analysis

All tests of significance were performed in R (version 4.4.1) using RStudio (version 2024.12.1 + 563). Pearson correlation coefficients, paired t-test, and ANOVA analysis were performed with the R Stats Package (version 4.4.1) (R Core Team, 2024). Herbicide injury resulted in an unequal number of surviving plants between herbicide doses, and thus Least-Square (LS) means were calculated for FB, PH True, and all digitally collected data parameters using the emmeans package (version 1.11.0) (Lenth, 2025). Data were fitted in a linear model including Treatment, Experimental Replication, Block, and their interactions as fixed effects. The interaction of Experimental Replication and Treatment was not significant (*P* > 0.05), indicating the Treatment effect was consistent across experimental replications, and the data can be pooled. Experimental Replication, Block, and their interactions were also non-significant (*P* > 0.05). Due to the lack of significance, the full model was compared to a reduced model including only Treatment as a fixed effect using a Likelihood Ratio Test, which indicated no significant difference in model fit (*P* > 0.05), and the reduced model yielded a lower Bayesian Information Criterion. Thus, the reduced model was utilized for LS mean calculation.

(1) $$Y_{i}=\;\beta_{0}+\;\beta_{1}Treatment\;+\;\epsilon_{i}\;\text{where}\;\epsilon_{i}∼N(0,\sigma^{2})$$

For this model, the Q-Q residuals indicated normality, but plots of residuals versus fitted values and scale-location indicated heteroscedasticity. A weighted linear regression approach was implemented to account for heteroscedasticity in the data, which improves robustness of *Y_i_* estimates by assigning lower weights to observations with higher residual variance and higher weights to observations with lower residual variance (Hooper, 1993; Rosopa et al., 2013). The residuals determined from Equation 1 were squared to determine a variance estimate, *v_i_*, for each observation (Equation 2).

(2) $$v_{i}={\left(Y_{i}-{\hat{Y}}_{i}\right)}^{2}$$

The weights for each observation, *w_i_*, were calculated as the inverse of these estimated variances (Equation 3).

(3) $$w_{i}=\frac{1}{v_{i}}$$

The data were fit to a new model that incorporated the weights (Equation 4).

(4) $$Y_{i}=\;\beta_{0}+\;\beta_{1}Treatment\;+\;\epsilon_{i}\;\text{where}\;\epsilon_{i}∼N(0,\frac{\sigma^{2}}{w_{i}})$$

The dose-response curves, GR_50_ estimates, and t-test comparisons of GR_50_ estimates were completed with the *drc* package (Version 3.0-1) (Ritz and Streibig, 2005; Knezevic et al., 2007). The three-parameter logistic regression model produced dose-response curves for morphological data, where *b* is the slope of the curve, *c* is the lower asymptote set to 0, *d* is the upper asymptote, *x* is the herbicide dose (g ai ha^-1^), and *y* is the LS mean of the morphological parameter expressed as a percentage of the untreated control. This model is more appropriate than Equation 6 due to the data for the morphological parameters ranging from 0 to ∞ (**Table 1**).

(5) $$y=\frac{d}{1+\text{exp}\{b[\log(x)-\log(GR_{50})]\}}$$

The four-parameter logistic regression model produced dose-response curves for spectral data, where *b* is the slope of the curve, *c* is the lower asymptote, *d* is the upper asymptote, *x* is the herbicide dose (g ai ha^-1^), and *y* is the LS mean of the spectral parameter expressed as a percentage of the untreated control. This model is more appropriate than Equation 5 due to the data for the spectral parameters ranging from -1 to 1 (**Table 1**).

(6) $$y=c+\frac{d-c}{1+\text{exp}\{b[\log(x)-\log(GR_{50})]\}}$$

## 3 Results

### 3.1 Correlation analysis and paired t-test

**Figure 1:**
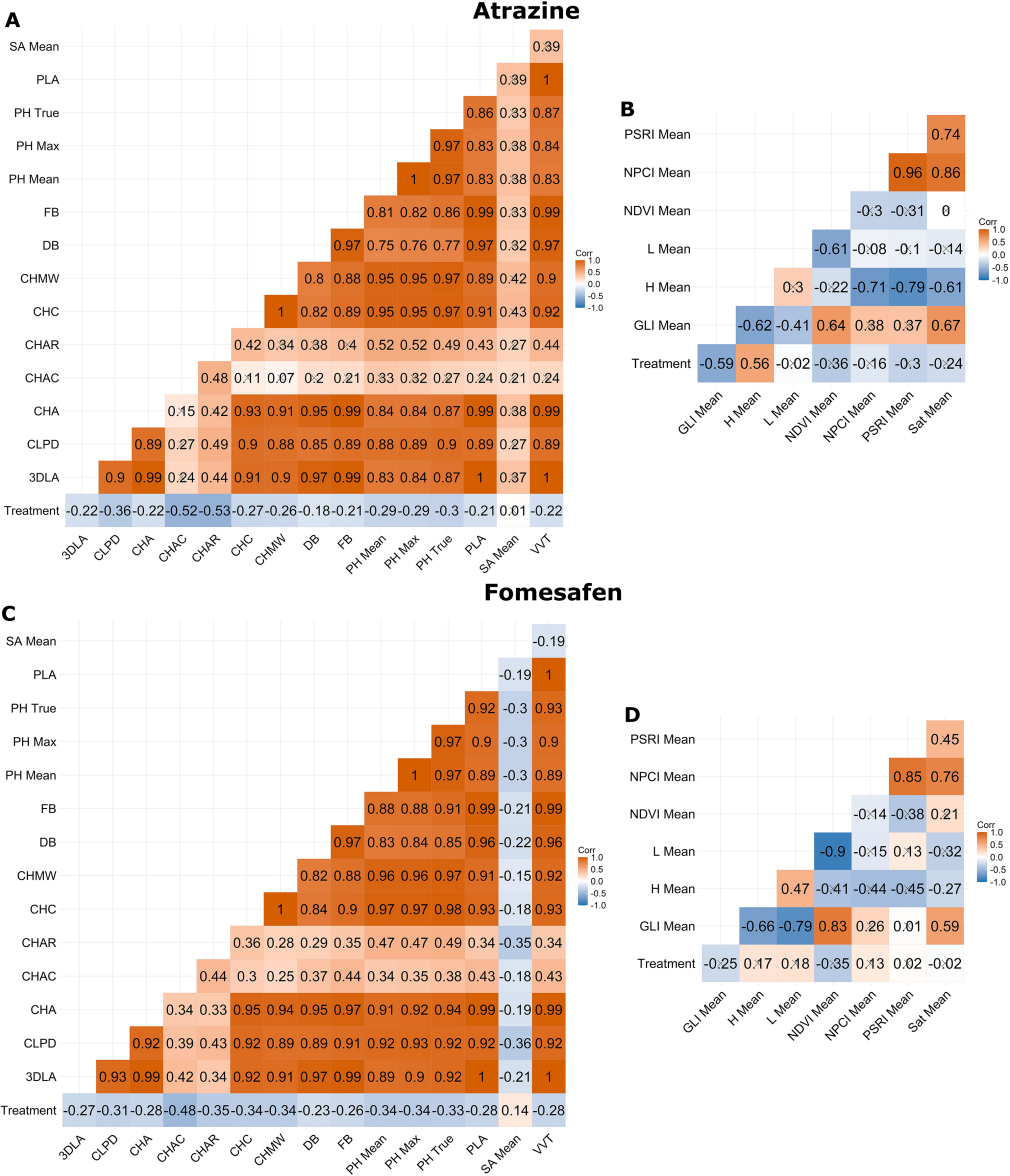
Pearson correlation coefficients between the specified herbicide treatment, manual measurements, morphological **(A, C)**, and spectral parameters **(B, D)**. Results for the atrazine experiment are indicated in **(A, B)** and results for the fomesafen experiment are indicated in **(C, D)**. Treatment represents increasing doses of the specified herbicide. An “X” in the background of the cell indicates *P* > 0.05.* A* lack of an “X” in the background of the cell indicates *P* < 0.05.

In both fomesafen and atrazine experiments, preliminary correlation analysis revealed strong positive correlations (*r* = 0.97, *P* < 0.05) occurred between digital parameters (DB, PH Max, and PH Mean) and their respective manual measurements (FB and PH True; **Figures 1A, C**). Except for Convex Hull Area Coverage, Convex Hull Aspect Ratio, and Surface Area Mean, morphological parameters displayed strong positive correlations (*r* ≥ 0.70, *P* < 0.05) with each other and the manual measurements (**Figures 1A, C**). Furthermore, some near-perfect and perfect positive correlations (*r* = 0.95 to *r* = 1.0, *P* < 0.05) were observed between DB, FB, 3DLA, Convex Hull Area, Projected Leaf Area, and Voxel Volume Total (CHA, PLA, and VVT, respectively; **Figures 1A, C**). Treatment displayed weak (*r* = -0.18, *P* < 0.05) to moderate (*r* = -0.53, *P* < 0.05) negative correlations for all manual measurements and morphological parameters, except for Surface Area Mean, which had zero to very weak (*r* = 0.14, *P* < 0.05) correlations among both experiments (**Figures 1A, C**).

Correlations between the spectral parameters and treatment were more variable between the atrazine and fomesafen experiments (**Figures 1B, D**). For the atrazine experiment, treatment had zero correlation with Lightness Mean and weak (*r* = -0.36 to -0.16, *P* > 0.05) to moderate (*r* = -0.59 or *r* = 0.56, *P* < 0.05) correlations with the remaining parameters (**Figure 1B**). In contrast, Plant Senescence Reflectance Index Mean and Saturation Mean had zero correlation with treatment, while the remaining parameters displayed weak (*r* = -0.35 to 0.18, *P* > 0.05) correlations in the fomesafen experiment (**Figure 1D**). Given that FB correlations with treatment ranged from *r* = -0.26 to -0.21 (*P* < 0.05), parameters with near-zero correlations (≥-0.1 or ≤0.1, *P* > 0.05) with treatment were not utilized for generating dose-response curves, since a near-zero correlation would suggest that parameters are not responding to the herbicide treatment and the data would not result in a sigmoidal dose-response curve that is comparable to a curve generated from FB data.

The manually and digitally collected PH data were compared using the paired t-test (**Table 2**). For both herbicides, the *P-*values indicate PH Max and PH Mean are significantly different from PH True (α = 0.05); however, the *P-*value associated with PH Max was consistently higher than the *P-*value associated with PH Mean (**Table 2**), indicating that it is a more accurate estimation of PH True. The smallest mean differences identified were approximately 0.67 mm and 1.05 mm for the atrazine and fomesafen experiments, respectively, indicating that the paired t-test had a high degree of power to detect significant differences between means.

**Table 2:** Summary paired t-test for plant height (PH) data. All PH data was recorded in millimeters.

Atrazine				
Parameter^†^	Mean (SE)^‡^			
PH True (mm)	12.59 (± 1.14)​			
PH Max (mm)	11.92 (± 1.08)​			
PH Mean (mm)	11.53 (± 1.04)​			
Paired t-test results				
Comparison	t-value	Degrees of freedom	Mean difference estimate	*P-*value
PH Max: PH True	-2.49​	287	-0.67	0.0135
PH Mean: PH True	-3.69	287	-1.06	0.0003

Fomesafen				
Parameter	Mean (SE)			
PH True (mm)	17.12 (± 1.50)​			
PH Max (mm)	16.06 (± 1.36)​			
PH Mean (mm)	15.34 (± 1.29)​			
Paired t-test results				
Comparison	t-value	Degrees of freedom	Mean difference estimate	*P-*value
PH Max: PH True	-2.97	287	-1.05	0.0032
PH Mean: PH True	-4.50	287	-1.77	9.9540 x 10^-6^

^†^PH True, manual measurements of plants; PH Max, digital measurement of the absolute highest point of the plant; PH Mean, digital measurement of the mean of the heights within the top 10% of the plant.

^‡^SE, standard error of the mean.

### 3.2 Dose-response curves and GR_50_ estimates

**Figure 2:**
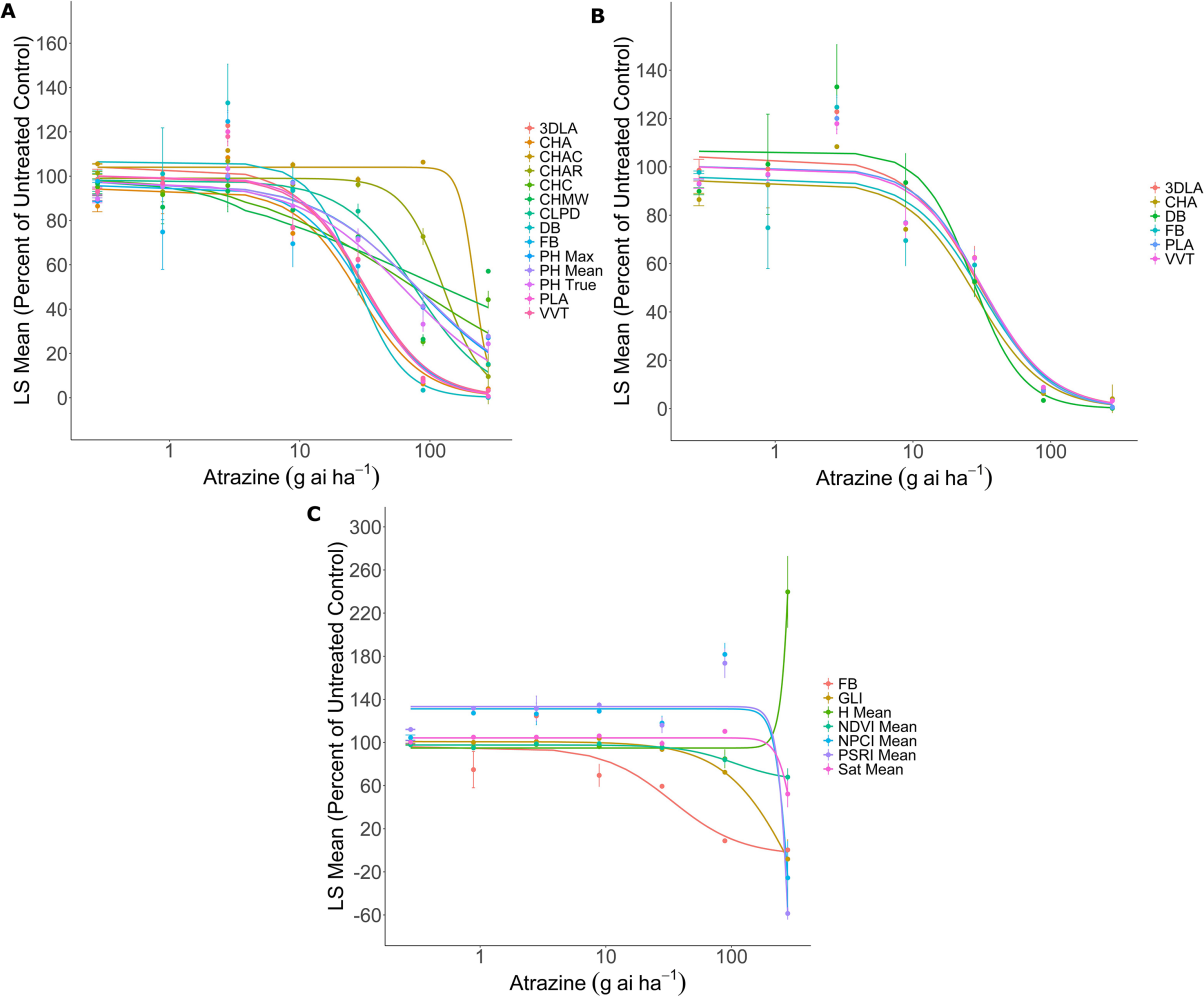
Atrazine dose-response curves generated from morphological **(A, B)** and spectral **(C)** parameters. Curves similar to the fresh biomass (FB) data are highlighted in **(B)**. Doses for atrazine ranged from 0.28 to 280 g ai ha^-1^ and were spaced by a factor of 3.16. Lines in **(A, B)** were fit with Equation 5: $y=\frac{d}{1+\text{exp}\{b[\log(x)-\log(GR_{50})]\}}$ and lines in (c) were fit with Equation 6: $y=c+\frac{d-c}{1+\text{exp}\{b[\log(x)-\log(GR_{50})]\}}$. Each circle represents the Least-Square (LS) mean expressed as a percentage of the untreated control. Error bars depict the standard error of the mean.

**Figure 3:**
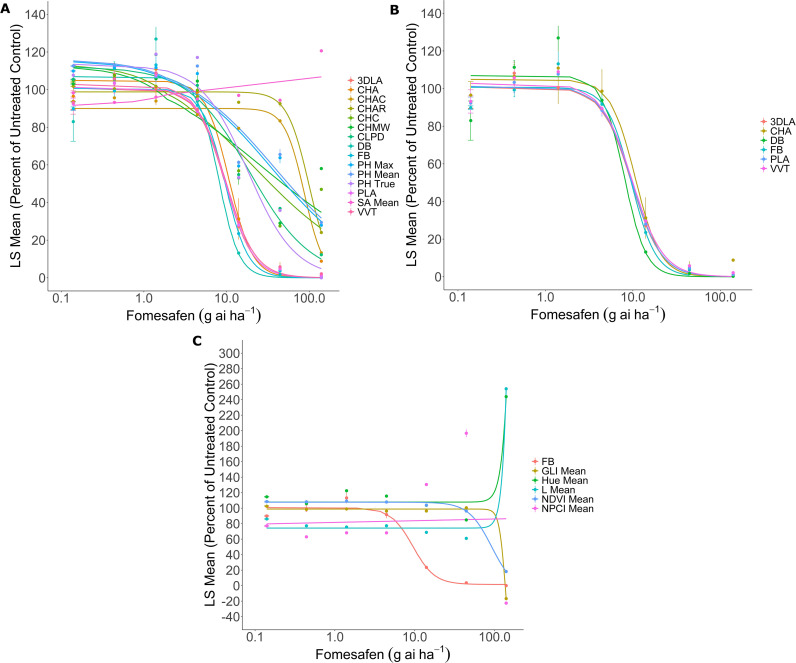
Fomesafen dose-response curves generated from morphological **(A, B)** and spectral **(C)** parameters. Curves similar to the fresh biomass (FB) data are highlighted in **(B)**. Doses for fomesafen ranged from 0.14 to 140 g ai ha^-1^ and were spaced by a factor of 3.16. Lines in **(A, B)** were fit with Equation 5: $y=\frac{d}{1+\text{exp}\{b[\log(x)-\log(GR_{50})]\}}$ and lines in **(C)** were fit with Equation 6: $y=c+\frac{d-c}{1+\text{exp}\{b[\log(x)-\log(GR_{50})]\}}$. Each circle represents the Least-Square (LS) mean expressed as a percentage of the untreated control. Error bars depict the standard error of the mean.

Using FB as the standard to which all results are compared, suitable digital parameters should generate visually similar dose-response curves and GR_50_ estimates that are not significantly different from estimates generated from FB data. The FB GR_50_ estimates were approximately 31.36 and 9.56 g ai ha^-1^ for atrazine and fomesafen, respectively, which is consistent with the knowledge that the current common lambsquarters population is sensitive to these herbicides. In both atrazine and fomesafen experiments, only 3DLA, CHA, DB, PLA, and VVT produced similar dose-response curves and GR_50_ estimates relative to FB (**Figures 2A, B**, **3A, B**); **Table 3**), indicating that these 5 parameters are accurate estimates of FB when utilizing atrazine and fomesafen on common lambsquarters. The remaining morphological parameters produce sigmoidal curves but yield GR_50_ estimates 2- to 10-fold higher than FB data (**Figures 2A**, **3A**; **Table 3**). Furthermore, the standard errors (SEs) associated with these GR_50_ estimates are high relative to SEs associated with FB (**Table 3**), which indicates the estimates are unreliable and not suitable for GR_50_ estimation.

**Table 3:** Summary of variables in the three-parameter logistic regression and growth reduction (GR_50_) estimates for morphological parameters.^†^

Atrazine				
Parameter^‡^	Model	GR_50_ estimate (SE)^§^ g ai ha^-1^	Parameter GR_50_:FB GR_50_ ratio (SE)	*P-*value for parameter GR_50_:FB GR_50_ ratio
3DLA	$y=\frac{104.16}{1+\text{exp}\left\{1.67\left[\log\left(x\right)-\log\left(29.57\right)\right]\right\}}$.	29.57​ (± 8.76)​*	0.94​ (± 0.40)​*	0.8887​*
CHA	$y=\frac{94.20}{1+\text{exp}1.76\left[\log\left(x\right)-\log\left(28.36\right)\right]\}}$	28.36​ (± 8.58)​*	0.90​ (± 0.39)​*	0.8085​*
CHAC	$y=\frac{104.04}{1+\text{exp}\left\{6.81\left[\log\left(x\right)-\log\left(215.37\right)\right]\right\}}$	215.37​ (± 112.56)​	6.87​ (± 4.18)​	0.1655​
CHAR	$y=\frac{99.05}{1+\text{exp}\left\{2.79\left[\log\left(x\right)-\log\left(126.91\right)\right]\right\}}$	126.91​ (± 28.93)	4.05​ (± 1.56)​	0.0558​
CHC	$y=\frac{100.34}{1+\text{exp}\left\{0.67\left[\log\left(x\right)-\log\left(74.18\right)\right]\right\}}$	74.18​ (± 43.61)​	2.37​ (± 1.57)​	0.3881​
CHMW	$y=\frac{104.35}{1+\exp\left\{0.44\left[\log\left(x\right)-\log\left(102.52\right)\right]\right\}}$	102.52 (± 112.87)	3.27​ (± 3.74)​	0.5465
CLPD	$y=\frac{98.14}{1+\text{exp}\left\{1.56\left[\log\left(x\right)-\log\left(77.17\right)\right]\right\}}$	77.17​ (± 22.46)​	2.46​ (± 1.05)​	0.1687
DB	$y=\frac{106.48}{1+\text{exp}\left\{2.39\left[\log\left(x\right)-\log\left(27.05\right)\right]\right\}}$	27.05​ (± 5.58)​*	0.86​ (± 0.32)​*	0.6713​*
FB	$y=\frac{95.67}{1+\text{exp}\left\{1.71\left[\log\left(x\right)-\log\left(31.36\right)\right]\right\}}$	31.36​ (± 9.75)​	NA​	NA​
PH Max	$y=\frac{97.44}{1+\text{exp}\left\{1.08\left[\log\left(x\right)-\log\left(81.65\right)\right]\right\}}$	81.65​ (± 31.44)​	2.60​ (± 0.19)​	0.0020
PH Mean	$y=\frac{97.65}{1+\text{exp}\left\{1.05\left[\log\left(x\right)-\log\left(82.49\right)\right]\right\}}$	82.49​ (± 32.24)​	2.63​ (± 0.19)​	0.0019​
PH True	$y=\frac{97.60}{1+\text{exp}\left\{1.07\left[\log\left(x\right)-\log\left(63.88\right)\right]\right\}}$	63.88​ (± 24.57)​	2.04​ (± 0.24)​	0.0404​
PLA	$y=\frac{100.07}{1+\text{exp}\left\{1.84\left[\log\left(x\right)-\log\left(31.74\right)\right]\right\}}$	31.74​ (± 9.04)​*	1.01​ (± 0.42)​*	0.9770​*
VVT	$y=\frac{99.98}{1+\text{exp}\left\{1.73\left[\log\left(x\right)-\log\left(31.96\right)\right]\right\}}$	31.96 ​(± 9.37)​*	1.02​ (± 0.42)​*	0.9643*

Fomesafen				
Parameter	Model	GR_50_ Estimate (SE) g ai ha^-1^	Parameter GR_50_:FB GR_50_ Ratio (SE)	*P-*value for Parameter GR_50_:FB GR_50_ Ratio
3DLA	$y=\frac{100.82}{1+\text{exp}\left\{2.54\left[\log\left(x\right)-\log\left(9.55\right)\right]\right\}}$	9.55​ (± 2.16)​*	1.00​ (± 0.32)​*	0.9977*
CHA	$y=\frac{104.88}{1+\text{exp}\left\{3.03\left[\log\left(x\right)-\log\left(10.65\right)\right]\right\}}$	10.65​ (± 2.21)​*	1.11​ (± 0.34)​*	0.7423*
CHAC	$y=\frac{90.00}{1+\text{exp}\left\{3.67\left[\log\left(x\right)-\log\left(87.09\right)\right]\right\}}$	87.09​ (± 24.42)​	9.11​ (± 3.30)​	0.0169
CHAR	$y=\frac{98.80}{1+\text{exp}\left\{3.29\left[\log\left(x\right)-\log\left(99.26\right)\right]\right\}}$	99.26​ (± 23.96)	10.38​ (± 3.46)​	0.0086
CHC	$y=\frac{116.29}{1+\text{exp}\left\{0.67\left[\log\left(x\right)-\log\left(22.32\right)\right]\right\}}$	22.32 (± 10.79)​	2.33​ (± 1.25)​	0.2895
CHMW	$y=\frac{118.60}{1+\text{exp}\left\{0.53\left[\log\left(x\right)-\log\left(26.40\right)\right]\right\}}$	26.40​ (± 17.78)​	2.76​ (± 1.96)​	0.3732
CLPD	$y=\frac{112.41}{1+\text{exp}\left\{1.17\left[\log\left(x\right)-\log\left(19.07\right)\right]\right\}}$	19.07​ (± 5.69)​	1.99 (± 0.75)​	0.1903
DB	$y=\frac{106.90}{1+\text{exp}\left\{3.51\left[\log\left(x\right)-\log\left(7.93\right)\right]\right\}}$	7.93 (± 1.83)​*	0.83​ (± 0.26)​*	0.5291*
FB	$y=\frac{100.69}{1+\text{exp}\left\{3.10\left[\log\left(x\right)-\log\left(9.56\right)\right]\right\}}$	9.56 (± 2.19)​	NA​	NA
PH Max	$y=\frac{116.35}{1+\text{exp}\left\{0.77\left[\log\left(x\right)-\log\left(35.34\right)\right]\right\}}$	35.34 (± 14.85)​	3.70​ (± 0.13)​	4.9463 x 10^-7^
PH Mean	$y=\frac{116.88}{1+\text{exp}\left\{0.76\left[\log\left(x\right)-\log\left(38.21\right)\right]\right\}}$	38.21​ (± 16.26)​	4.00 (± 0.12)​	5.6220 x 10^-8^
PH True	$y=\frac{113.54}{1+\text{exp}\left\{1.52\left[\log\left(x\right)-\log\left(18.05\right)\right]\right\}}$	18.05​ (± 4.57)​	1.89​ (± 0.18)​	0.0117
PLA	$y=\frac{101.20}{1+\text{exp}\left\{2.55\left[\log\left(x\right)-\log\left(9.64\right)\right]\right\}}$	9.64​ (± 2.15)​*	1.01​ (± 0.32)​*	0.9785*
SA Mean	$y=\frac{209.50}{1+\text{exp}\left\{-0.04\left[\log\left(x\right)-\log\left(55.59\right)\right]\right\}}$	55.59 ​(± 1521.43)​	5.81​ (± 0.42)	0.8610
VVT	$y=\frac{102.77}{1+\text{exp}\left\{2.44\left[\log\left(x\right)-\log\left(9.75\right)\right]\right\}}$	9.75 ​(± 2.14)​*	1.02 (± 0.31)​*	0.9516*

^†^For each experiment, parameters that generated dose-response curves and GR50 estimates similar to FB data are highlighted with ‘*’.

^‡^3DLA, 3D leaf area; CHA, convex hull area; CHAC, convex hull area coverage; CHAR, convex hull aspect ratio; CHC, convex hull circumference; CHMW, convex hull max width; CLPD, canopy light penetration depth; DB, digital biomass; FB, fresh biomass; PH Max, plant height max; PH Mean, plant height mean; PH True, plant height true; PLA, projected leaf area; SA Mean, surface area mean; VVT, voxel volume total.

^§^SE, standard error of the mean.

In contrast, the dose-response curves of the spectral data are very dissimilar from the FB curve, and they generally indicate little variation across the treatments except at the two highest doses (**Figures 2C**, **3C**). The resulting GR_50_ estimates are 7- to 24-fold higher than FB with very high SEs, indicating unreliable GR_50_ estimation (**Table 4**).

**Table 4:** Summary of variables in the four-parameter logistic regression of growth reduction (GR_50_) estimates for spectral parameters.^†^

Atrazine					
Parameter^‡^	Model	GR_50_ estimate (SE)^§^ g ai ha^-1^	Parameter GR_50_:FB GR_50_ ratio (SE)	*P-*value for parameter GR_50_:FB GR_50_ ratio	
FB	$y=-5.37+\frac{96.11--5.37}{1+\text{exp}\left\{1.56\left[\log\left(x\right)-\log\left(33.99\right)\right]\right\}}$	33.99​ (± 20.70)​	NA​	NA​	
GLI	$y=-278.49+\frac{100.99--278.49}{1+\text{exp}\left\{1.39\left[\log\left(x\right)-\log\left(537.93\right)\right]\right\}}$	537.93​ (± 17,061.94)​	17.15 (± 2.00)​	0.6451	
H Mean	$y=94.90+\frac{1014.85-94.90}{1+\text{exp}\left\{-9.79\left[\log\left(x\right)-\log\left(332.33\right)\right]\right\}}$	332.33​ (± 147.36)	10.60​ (± 0.08)​	1.2501 x 10^-10^	
NDVI Mean	$y=-14.22+\frac{98.26--14.22}{1+\text{exp}\left\{0.96\left[\log\left(x\right)-\log\left(772.33\right)\right]\right\}}$	772.33​ (± 307.11)​	24.63 (± 0.15)​	1.0680 x 10^-6^	
NPCI Mean	$y=-2082.85+\frac{131.21--2082.85}{1+\text{exp}\left\{10.10\left[\log\left(x\right)-\log\left(361.22\right)\right]\right\}}$	361.22​ (± 1913.63)​	11.52 (± 0.5)​	0.0854	
PSRI Mean	$y=1860.86+\frac{133.34--1860.86}{1+\text{exp}\left\{10.16\left[\log\left(x\right)-\log\left(349.07\right)\right]\right\}}$	349.07 (± 123.81)​	11.13​ (± 0.02)​	2.2051 x 10^-16^	
Sat Mean	$y=-862.37+\frac{104.29--862.37}{1+\text{exp}\left\{8.74\left[\log\left(x\right)-\log\left(388.69\right)\right]\right\}}$	388.69​ (± 856.12)​	12.39​ (± 0.20)​	0.0002	

Fomesafen					
Parameter	Model	GR_50_ Estimate (SE) g ai ha^-1^	Parameter GR_50_:FB GR_50_ Ratio (SE)		*P-*value for Parameter GR_50_:FB GR_50_ Ratio
FB	$y=18.34+\frac{100.67-18.34}{1+\text{exp}\left\{3.11\left[\log\left(x\right)-\log\left(GR_{50}\right)\right]\right\}}$	9.55 (± 8.05)​	NA​		NA
GLI	$y=-155.12+\frac{98.79--155.12}{1+\text{exp}\left\{10.34\left[\log\left(x\right)-\log\left(GR_{50}\right)\right]\right\}}$	142.49​ (± 51.02)​	14.90 (± 0.05)​		8.2201 x 10^-13^
H Mean	$y=107.80+\frac{1256.10-107.80}{1+\text{exp}\left\{-10.21\left[\log\left(x\right)-\log\left(GR_{50}\right)\right]\right\}}$	170.42​ (± 99.29)	17.83​ (± 0.01)​		2.2051 x 10^-16^
L Mean	$y=74.28+\frac{1343.10-74.28}{1+\text{exp}\left\{-8.46\left[\log\left(x\right)-\log\left(GR_{50}\right)\right]\right\}}$	171.82 (± 131.02)​	17.97 (± 0.06)​		1.360 x 10^-11^
NDVI Mean	$y=-89.54+\frac{395.81--89.54}{1+\text{exp}\left\{2.22\left[\log\left(x\right)-\log\left(GR_{50}\right)\right]\right\}}$	152.14​ (± 2257.90)​	15.91 (± 0.93)​		0.3283
NPCI Mean	$y=-229.90+\frac{171.36--229.90}{1+\text{exp}\left\{-22.163\left[\log\left(x\right)-\log\left(GR_{50}\right)\right]\right\}}$	75.23 (± 9.99)​	7.88​ (± 0.01)​		9.2218 x 10^-8^

^†^†For each experiment, parameters that generated results similar to FB are highlighted with ‘*’.

^‡^FB, fresh biomass; GLI, green leaf index; H Mean, hue mean; L Mean, lightness mean; NDVI Mean, normalized difference vegetation index mean; NPCI Mean, normalized pigment chlorophyll index mean; PSRI Mean, plant senescence reflectance index mean; Sat Mean, saturation mean.

^§^SE, standard error of the mean.

## 4 Discussion

Correlations between DB and FB in current experiments were strong (R^2^ = 0.94, *r* = 0.97, *P* < 0.05), which is reflective of previous experiments in other species where correlations of DB to dry or fresh biomass were also quite strong in *T. durum* and *S. cereale* (R^2^ = 0.85; *r* = 0.92) (Bazhenov et al., 2023) and hemp (*r* = 0.89) (Singh et al., 2024). Furthermore, the generally strong correlations observed among morphological parameters and manual measurements (**Figure 1**) were expected and demonstrate how the Phenospex TraitFinder was designed to be readily applied for non-destructive phenotyping of common morphological traits (Manavalan et al., 2021; Schafleitner et al., 2021; Miroshnichenko et al., 2022; Bazhenov et al., 2023; Mirgorodskaya et al., 2023; Quijia Pillajo et al., 2023, Quijia Pillajo et al., 2024; Zieschank and Junker, 2023; Galba et al., 2025; Mohammed et al., 2025; Quijia-Pillajo and Jones, 2025). Due to the negative, sigmoidal relationship that is typical of dose-response curves (Seefeldt et al., 1995; Knezevic et al., 2007; Keshtkar et al., 2021), relatively weak, negative correlations (*r* = -0.26 to -0.21) between treatment and FB were expected (**Figures 1A, C**). By using these results as a guideline, there were multiple parameters with similar correlations to treatment (**Figure 1**) and thus could potentially produce sigmoidal curves similar to FB data. While correlation analysis can identify the type and strength of a linear relationship between two variables, it does not indicate the accuracy of the data or if the data is suitable for estimating a GR_50_, and consequently, other means of evaluation were needed. Since both manually and digitally collected PH measurements were recorded in mm (**Table 1**), these results could be compared with a paired t-test in addition to comparing the results of the dose-response curves and GR_50_ estimates. In contrast, DB and FB are measured in mm^3^ and milligrams, respectively, and therefore, comparing the results of the dose-response curves and GR_50_ estimates was more appropriate.

The few previous investigations involving the Phenospex TraitFinder and comparisons of manual and digital PH measurements have also reported high positive correlations of *r* = 0.74 to 0.83 in *Petunia × hybrida* (Quijia Pillajo et al., 2023) and *r* = 0.94 in hemp (Singh et al., 2024), but no other comparisons of these data were performed. High correlations between manually and digitally collected PH measurements were expected (**Table 1**) and demonstrated in current experiments (*r* = 0.97, *P* < 0.05; **Figure 1**). Further analysis with the paired t-test consistently indicated that the measurements for PH Max and PH Mean were significantly different from PH True (**Table 2**). However, the higher *P-*values associated with PH Max indicate it was consistently the more accurate measurement for overall plant height. This result is likely due to PH Max being measured from the base of the plant to its highest point rather than the average of the highest 10% of points as with PH Mean (**Table 1**). The methods for the PH Max measurements are more comparable to methods employed for PH True and other experiments measuring overall plant height (Gao et al., 2015; Zhang et al., 2019; Quijia Pillajo et al., 2023; Boatwright et al., 2024).

Despite the consistent differences identified between PH True and PH Mean by the paired t-test, these differences did not influence DB data. In both experiments, dose-response curves and GR_50_ estimates generated from DB data did not significantly differ from FB results (**Table 3**; **Figures 2A, B**, **3A, B**), indicating DB accurately reflects FB data. There could be scenarios where accurate measuring of PH data requires very high accuracy; however, for the purposes of collecting DB for dose-response curve generation in common lambsquarters, the accuracy of PH Mean data is sufficient, and the differences detected by the paired t-test do not impact DB as an indirect estimator for FB. It is also worth noting that the power of paired t-test is determined by the level of correlation between measurements (Zweifach, 2024), which was consistently high for both experiments (*r* = 0.97, *P* < 0.05). The high correlations and high sample size allow for the power of the current paired t-test to be great enough to identify small differences in PH measurements as significant (**Table 2**). While having high statistical power is generally the goal when designing experiments, current results demonstrate how having the power to detect small statistical differences does not necessarily equate to biological significance (Lovell, 2013; Alexander and Davis, 2022; Zweifach, 2024).

For dose-response curve generation, estimation of GR_50_ and GR_50_ SEs indicated that only morphological parameters, such as 3DLA, CHA, DB, PLA, and VVT, produce similar results relative to FB data in both experiments (**Table 3**; **Figures 2A, B**, **3A, B**), indicating any one or all of the five parameters could be utilized to generate dose-response curves and estimate GR_50_ values for fomesafen- and atrazine-treated common lambsquarters. In contrast, none of the spectral parameters are suitable for dose-response curve generation. Current results indicate that at 21 DAT, biomass from surviving common lambsquarters seedlings will decrease as herbicide dose increases, but the spectral data indicate there is little change in plant stress levels relative to the untreated plants except at the two highest doses of the herbicide (**Figures 2C**, **3C**). While previous research with the Phenospex TraitFinder demonstrates the ability to find differences in the spectral data among different herbicide formulations (Mirgorodskaya et al., 2023) as well as other treatment conditions (Bazhenov et al., 2023; Zieschank and Junker, 2023; Quijia Pillajo et al., 2024; Quijia-Pillajo and Jones, 2025), most of the herbicide doses implemented in current experiments do not produce large changes in the spectral data except at the highest doses (**Figures 2C**, **3C**). However, it should be noted that results for spectral data would have yielded different insights if data were collected at earlier timepoints, such as 3 to 14 DAT, when injury symptoms would have been more apparent across all doses. One of the benefits of the Phenospex TraitFinder is that data can be collected throughout the experiment, and it is well established that spectral data will vary throughout plant development (Miroshnichenko et al., 2022; Quijia Pillajo et al., 2024; Quijia-Pillajo and Jones, 2025) and stress injury progression (Schafleitner et al., 2021; Mirgorodskaya et al., 2023). Furthermore, previous experiments have demonstrated the utility of multispectral data for phenotyping herbicide injury over time (Huang et al., 2015; Da Silva et al., 2019), distinguishing between resistant and sensitive biotypes, especially at earlier points during an experiment (Xia et al., 2022; Jones et al., 2023b, Jones et al., 2023a). Thus, it is worthwhile to further explore the utility of spectral data collected by the Phenospex TraitFinder in experiments where data will be collected throughout the duration of the experiment. For current experiments, our focus was on the utility and accuracy of digital data when the Phenospex TraitFinder is implemented in an experiment that reflects commonly implemented dose-response practices, where destructive biomass data collection occurs at the conclusion of the experiment.

Whether the same trends for morphological and spectral parameters will be observed for common lambsquarters dose-response assays utilizing other herbicides with different modes of action warrants further exploration. Furthermore, examining other dicots and grass species will also be necessary in the future since morphological features that greatly differ from common lambsquarters would likely result in different trends in suitable parameters. Based on available information about the consistency of parameter correlations with plant biomass (**Table 1**), we hypothesize that the digital parameters (3DLA, DB, and PLA) will consistently generate accurate dose-response curves and GR_50_ estimates regardless of species or herbicide, but the technical parameters (CHA and VVT) will likely vary by species and herbicide.

The Phenospex TraitFinder provides multiple metrics for estimating the GR_50_, including 3DLA, CHA, DB, PLA, and VVT (**Table 3**). Implementing some form of digital phenotyping allows researchers to decrease turnaround time to complete phenotyping experiments and share relevant findings. Furthermore, by not sacrificing plants to collect biomass data, these plants can be utilized for other purposes, such as phenotyping for traits displayed at later growth stages, extraction of protein or nucleic acids, and seed increase. Increasing seed of resistant and sensitive biotypes is of interest since the progeny can be utilized for making crosses that would be valuable in other phenotyping experiments, like genome-wide association studies or bulk segregant analysis, which can be performed with a segregating F_2_ population (Taylor et al., 2018; Majeed et al., 2022). The phenotyping required for these experiments is a practical application of a digital phenotyping system since phenotyping of numerous plants is essential (Uffelmann et al., 2021; Majeed et al., 2022). These experiments are becoming more desirable as researchers endeavor to identify the multiple genes underpinning non-target site resistance mechanisms in weed species (Liu et al., 2019; Patterson et al., 2019; Murphy et al., 2021; Brunharo et al., 2025), such as enhanced metabolism, reduced absorption, altered translocation, and rapid necrosis (Gaines et al., 2020).

## Data Availability

The datasets presented in this study can be found in online repositories. The names of the repository/repositories and accession number(s) can be found below: 10.15482/USDA.ADC/29815082 or https://figshare.com/s/64d1bbac59a95c4721f1.

## Supplementary material

Supplementary Figure S1 The Phenospex TraitFinder utilized for this research. Pots were arranged in a 3x6 pattern and elevated to a height of 395 mm.

Supplementary Figure S2 An example of HortControl block configuration. Pots were arranged in a 3x6 grid, with each cell of the grid containing one pot from which data was collected. A color filter for normalized difference vegetation index is currently implemented to make the plants and pots more visible during block configuration. The color filter was not utilized for data collection.

Supplementary Figure S3 An example setup for the training-based color filter using the same plants from **Supplementary Figure 2**. Part of the randomized complete block design involved arranging pots in a 3x6 patten that included two blank spots to serve as a negative control for PlantEye F600 3D scanners. Figure **(A)** depicts the red-green-blue image acquired by the PlantEye F600 3D scanners. Ten blue dots are assigned to plant material and ten red dots are assigned to non-plant material (e.g. soil, pot edges, perlite, etc.) to train HortControl, which is depicted in Figure (b). Figure **(C)** shows HortControl’s initial guess based on the input from Figure **(B)**. Figure **(D)** displays additional dots being added to improve HortControl training. Figure **(E)** indicates successful differentiation between plant and non-plant material by HortControl. Note that HortControl is detecting two small seedlings located in third row of the 5^th^ and 6^th^ columns, which were not easily apparent in **Supplementary Figure 2**.

## References

[B1] Alexander B. C. S. Davis A. S. (2022). Perspective: scientific rigor or ritual? Statistical significance in pest management science. Pest Manage. Sci. 78, 847–854. doi: 10.1002/ps.6668 34599550

[B2] Anderson M. Hartzler B. (2020). Identifying common herbicide symptoms in soybean ( Iowa State University Extension and Outreach). Available online at: https://crops.extension.iastate.edu/post/identifying-common-herbicide-symptoms-soybean (Accessed December 27, 2025).

[B3] Anonymous (2022). Aatrex® 4L herbicide product label. Available online at: https://www.cdms.net/ldat/ld6BJ021.pdf (Accessed December 27, 2025).

[B4] Anonymous (2024). Reflex® herbicide product label. Available online at: https://www.cdms.net/ldat/ld6BJ021.pdf (Accessed December 27, 2025).

[B5] Bazhenov M. Litvinov D. Karlov G. Divashuk M. (2023). Evaluation of phosphate rock as the only source of phosphorus for the growth of tall and semi-dwarf durum wheat and rye plants using digital phenotyping. PeerJ 11, e15972. doi: 10.7717/peerj.15972 37663276 PMC10473039

[B6] Boatwright L. Thudi M. Sangireddy M. K. R. Coffin A. W. Tadesse H. K. Vutla S. . (2024). GWAS analysis for plant height and stem diameter in sorghum using multiple phenotyping approaches. Plant Phenome. J. 7, e70008. doi: 10.1002/ppj2.70008 41859965

[B7] Borgato E. A. Thiagarayaselvam A. Peterson D. E. Hay M. M. Dille J. A. Jugulam M. (2024). Metabolic resistance to protoporphyrinogen oxidase-inhibitor herbicides in a Palmer amaranth population from Kansas. J. Agric. Food. Chem. 72, 5122–5132. doi: 10.1021/acs.jafc.3c05333 38382533

[B8] Brunharo C. A. C. G. Hanson B. D. (2018). Multiple herbicide-resistant Italian ryegrass (Lolium perenne L. spp. multiflorum (Lam.) Husnot) in California perennial crops: characterization, mechanism of resistance, and chemical management. Weed. Sci. 66, 696–701. doi: 10.1017/wsc.2018.50 41822556

[B9] Brunharo C. A. Short A. W. Bobadilla L. K. Streisfeld M. A. (2025). The genome of Lolium multiflorum reveals the genetic architecture of paraquat resistance. Mol. Ecol. 34, e17775. doi: 10.1111/mec.17775 40285737 PMC12051776

[B10] Burgos N. R. Tranel P. J. Streibig J. C. Davis V. M. Shaner D. Norsworthy J. K. . (2013). Confirmation of resistance to herbicides and evaluation of resistance levels. Weed. Sci. 61, 4–20. doi: 10.1614/ws-d-12-00032.1

[B12] Da Silva A. R. De Freitas M. A. M. De Souza Costa D. Da Silva Araújo L. De Almeida Rocha R. Dos Santos P. V. . (2019). Proximal sensing estimation of glyphosate injury on weeds in central Brazil. J. Appl. Remote Sens. 13, 44524. doi: 10.1117/1.jrs.13.044524 41586156

[B14] Gaines T. A. Duke S. O. Morran S. Rigon C. A. G. Tranel P. J. Kuepper A. . (2020). Mechanisms of evolved herbicide resistance. J. Biol. Chem. 295, 10307–10330. doi: 10.1074/jbc.REV120.013572 32430396 PMC7383398

[B13] Galba A. Masner J. Kholová J. Kartal S. Stočes M. Mikeš V. . (2025). Annotated 3D point cloud dataset of broad-leaf legumes captured by high-throughput phenotyping platform. Sci. Data 12, 1764. doi: 10.1038/s41597-025-06049-7 41213977 PMC12603112

[B15] Gao F. Wen W. Liu J. Rasheed A. Yin G. Xia X. . (2015). Genome-wide linkage mapping of QTL for yield components, plant height and yield-related physiological traits in the Chinese wheat cross Zhou 8425B/Chinese spring. Front. Plant Sci. 6. doi: 10.3389/fpls.2015.01099 26734019 PMC4683206

[B16] Hooper P. M. (1993). Iterative weighted least squares estimation in heteroscedastic linear models. J. Am. Stat. Assoc. 88, 179–184. doi: 10.1080/01621459.1993.10594309 41858497

[B17] Huang Y. Reddy K. N. Thomson S. J. Yao H. (2015). Assessment of soybean injury from glyphosate using airborne multispectral remote sensing. Pest Manage. Sci. 71, 545–552. doi: 10.1002/ps.3839 24889377

[B18] Jones E. A. L. Austin R. Dunne J. C. Cahoon C. W. Jennings K. M. Leon R. G. . (2023a). Utilization of image-based spectral reflectance to detect herbicide resistance in glufosinate-resistant and glufosinate-susceptible plants: A proof of concept. Weed. Sci. 71, 11–21. doi: 10.1017/wsc.2022.68 41822556

[B19] Jones E. A. L. Austin R. Dunne J. C. Leon R. G. Everman W. J. (2023b). Discrimination between protoporphyrinogen oxidase-inhibiting herbicide-resistant and herbicide-susceptible redroot pigweed (Amaranthus retroflexus) with spectral reflectance. Weed. Sci. 71, 198–205. doi: 10.1017/wsc.2023.25 41822556

[B20] Keshtkar E. Kudsk P. Mesgaran M. B. (2021). Perspective: common errors in dose–response analysis and how to avoid them. Pest Manage. Sci. 77, 2599–2608. doi: 10.1002/ps.6268 33415846

[B21] Knezevic S. Z. Streibig J. C. Ritz C. (2007). Utilizing R software package for dose-response studies: the concept and data analysis. Weed. Sci. 21, 840–848. doi: 10.1614/WT-06-161.1

[B22] Lenth R. V. (2025). “ emmeans: estimated marginal means, aka least-squares means,” in R package version 1.11.0. Available online at: https://CRAN.R-project.org/package=emmeans.

[B23] Liu X. Bi B. Xu X. Li B. Tian S. Wang J. . (2019). Rapid identification of a candidate nicosulfuron sensitivity gene (Nss) in maize (Zea mays L.) via combining bulked segregant analysis and RNA-seq. Theo. Appl. Genet. 132, 1351–1361. doi: 10.1007/s00122-019-03282-8 30652203

[B24] Lovell D. P. (2013). Biological importance and statistical significance. J. Agric. Food. Chem. 61, 8340–8348. doi: 10.1021/jf401124y 23909755

[B25] Lu H. Yu Q. Han H. Owen M. J. Powles S. B. (2020). Evolution of resistance to HPPD-inhibiting herbicides in a wild radish population via enhanced herbicide metabolism. Pest Manage. Sci. 76, 1929–1937. doi: 10.1002/ps.5725 31854080

[B26] Ma R. Evans A. F. Riechers D. E. (2016). Differential responses to preemergence and postemergence atrazine in two atrazine-resistant waterhemp populations. Agron. J. 108, 1196–1202. doi: 10.2134/agronj2015.0571

[B27] Majeed A. Johar P. Raina A. Salgotra R. K. Feng X. Bhat J. A. (2022). Harnessing the potential of bulk segregant analysis sequencing and its related approaches in crop breeding. Front. Genet. 13. doi: 10.3389/fgene.2022.944501 36003337 PMC9393495

[B28] Manavalan L. P. Cui I. Ambrose K. V. Panjwani S. DeLong S. Mleczko M. . (2021). Systematic approach to validate and implement digital phenotyping tool for soybean: a case study with PlantEye. Plant Phenome. J. 4, e20025. doi: 10.1002/ppj2.20025 41859965

[B29] Mirgorodskaya A. B. Kushnazarova R. A. Zakharova L. Y. Ulyanova A. A. Litvinov D. Y. Blinkov A. O. . (2023). Enhanced herbicidal action of clopyralid in the form of a supramolecular complex with a gemini surfactant. Agronomy 13, 973. doi: 10.3390/agronomy13040973 41725453

[B30] Miroshnichenko D. Timerbaev V. Klementyeva A. Pushin A. Sidorova T. Litvinov D. . (2022). CRISPR/Cas9-induced modification of the conservative promoter region of VRN-A1 alters the heading time of hexaploid bread wheat. Front. Plant Sci. 13. doi: 10.3389/fpls.2022.1048695 36544871 PMC9760837

[B31] Mohammed S. P. Yen J.-Y. Hsu Y.-C. Chou H.-Y. Natarajan S. Eybishitz A. (2025). Integrative trait analysis for enhancing heat stress resilience in tomato (Solanum lycopersicum L.): a focus on root, physiological, and yield adaptations. Plants 14, 533. doi: 10.3390/plants 40006792 PMC11858947

[B32] Murphy B. P. Beffa R. Tranel P. J. (2021). Genetic architecture underlying HPPD-inhibitor resistance in a Nebraska Amaranthus tuberculatus population. Pest Manage. Sci. 77, 4884–4891. doi: 10.1002/ps.6560 34272808

[B33] Obenland O. A. Ma R. O’Brien S. R. Lygin A. V. Riechers D. E. (2019). Carfentrazone-ethyl resistance in an Amaranthus tuberculatus population is not mediated by amino acid alterations in the PPO2 protein. PloS One 14, e0215431. doi: 10.1371/journal.pone.0215431 30986256 PMC6464220

[B34] Patterson E. L. Saski C. Küpper A. Beffa R. Gaines T. A. (2019). Omics potential in herbicide-resistant weed management. Plants 8, 607. doi: 10.3390/plants8120607 31847327 PMC6963460

[B11] Phenospex (2021). “ Comparing PlantEye F600 vs. F500,” in Phenospex. Available online at: https://phenospex.com/blog/comparing-planteye-f600-vs-f500/.

[B35] Phenospex (2024). Hortcontrol 3.14 manual 2024 Phenospex.

[B36] Phenospex (2025a). PlantEye F600 product data sheet version 1.1.0 2025 Phenospex.

[B37] Phenospex (2025b). TraitFinder F600 product data sheet version 1.0.0 2025 Phenospex.

[B38] Priess G. L. Norsworthy J. K. Godara N. Mauromoustakos A. Butts T. R. Roberts T. L. . (2022). Confirmation of glufosinate-resistant Palmer amaranth and response to other herbicides. Weed. Technol. 36, 368–372. doi: 10.1017/wet.2022.21 41822556

[B39] Quijia Pillajo J. Chapin L. J. Martins E. M. Jones M. L. (2024). A biostimulant containing humic and fulvic acids promotes growth and health of tomato ‘Bush Beefsteak’ plants. Horticulturae 10, 671. doi: 10.3390/horticulturae10070671 41725453

[B40] Quijia Pillajo J. Chapin L. Naik S. Jones M. L. (2023). Sustainable production of greenhouse ornamentals using plant growth-promoting bacteria. Acta Hortic. 1383, 99–108. doi: 10.17660/ActaHortic.2023.1383.11

[B41] Quijia-Pillajo J. Jones M. L. (2025). Lalrise Vita improves performance of French marigolds grown at pH 7.0 and fertilized with calcium phosphate. HortScience 60, 684–686. doi: 10.21273/HORTSCI18514-25

[B43] Randell-Singleton T. Hand L. C. Vance J. C. Wright-Smith H. E. Culpepper A. S. (2024). Confirming resistance to PPO-inhibiting herbicides applied preemergence and postemergence in a Georgia Palmer amaranth population. Weed. Technol. 38:1–10. doi: 10.1017/wet.2024.12 41822556

[B44] Rangani G. Noguera M. Salas-Perez R. Benedetti L. Roma-Burgos N. (2021). Mechanism of resistance to S-metolachlor in Palmer amaranth. Front. Plant Sci. 12. doi: 10.3389/fpls.2021.652581 33777086 PMC7994610

[B42] R Core Team (2024). R: a language and environment for statistical computing. Available online at: https://www.R-project.org/ (Accessed December 27, 2025).

[B45] Ribeiro V. H. V. Brunharo C. A. C. G. Mallory-Smith C. Walenta D. L. Barroso J. (2023). First report of target-site resistance to ACCase-inhibiting herbicides in Bromus tectorum L. Pest Manage. Sci. 79, 4025–4033. doi: 10.1002/ps.7607 37309712

[B46] Ritz C. Streibig J. C. (2005). Bioassay analysis using R. J. Stat. Software 12, 1–22. doi: 10.18637/jss.v012.i05

[B47] Rodriguez J. Hauvermale A. Carter A. Zuger R. Burke I. C. (2021). An ALA122THR substitution in the AHAS/ALS gene confers imazamox-resistance in Aegilops cylindrica. Pest Manage. Sci. 77, 4583–4592. doi: 10.1002/ps.6498 34087037

[B48] Rosopa P. J. Schaffer M. M. Schroeder A. N. (2013). Managing heteroscedasticity in general linear models. Psychol. Methods 18, 335–351. doi: 10.1037/a0032553 24015776

[B49] Schafleitner R. Lin C. Y. Lin Y. P. Wu T. H. Hung C. H. Phooi C. L. . (2021). The world vegetable center okra (Abelmoschus esculentus) core collection as a source for flooding stress tolerance traits for breeding. Agriculture 11, 165. doi: 10.3390/agriculture11020165 41725453

[B50] Schwartz-Lazaro L. M. Norsworthy J. K. Scott R. C. Barber L. T. (2017). Resistance of two Arkansas Palmer amaranth populations to multiple herbicide sites of action. Crop Prot. 96, 158–163. doi: 10.1016/j.cropro.2017.02.022 41862359

[B51] Seefeldt S. S. Jensen J. E. Fuerst E. P. (1995). Log-logistic analysis of herbicide dose-response relationships. Weed. Technol. 9, 218–227. doi: 10.1017/s0890037x00023253 41822556

[B52] Shrestha S. Sharma G. Burgos N. R. Tseng T. M. (2019). Response of weedy rice (Oryza spp.) germplasm from Arkansas to glyphosate, glufosinate, and flumioxazin. Weed. Sci. 67, 303–310. doi: 10.1017/wsc.2018.92 41822556

[B53] Shyam C. Peterson D. E. Jugulam M. (2022). Resistance to 2,4-D in Palmer amaranth (Amaranthus palmeri) from Kansas is mediated by enhanced metabolism. Weed. Sci. 70, 390–400. doi: 10.1017/wsc.2022.29 41822556

[B54] Singh G. Slonecki T. Wadl P. Flessner M. Sosnoskie L. Hatterman-Valenti H. . (2024). Implementing digital multispectral 3D scanning technology for rapid assessment of hemp (Cannabis sativa L.) weed competitive traits. Remote Sens. 16, 2375. doi: 10.3390/rs16132375 41725453

[B55] Strom S. A. Gonzini L. C. Mitsdarfer C. Davis A. S. Riechers D. E. Hager A. G. (2019). Characterization of multiple herbicide-resistant waterhemp (Amaranthus tuberculatus) populations from Illinois to VLCFA-inhibiting herbicides. Weed. Sci. 67, 369–379. doi: 10.1017/wsc.2019.13 41822556

[B56] Székács A. (2021). “ Herbicide mode of action,” in Herbicides: Chemistry, Efficacy, Toxicology, and Environmental Impacts (Amsterdam, Netherlands: Elsevier), 41–86. doi: 10.1016/B978-0-12-823674-1.00008-0

[B57] Taylor M. Tornqvist C. E. Zhao X. Grabowski P. Doerge R. Ma J. . (2018). Genome-wide association study in pseudo-F_2_ populations of switchgrass identifies genetic loci affecting heading and anthesis dates. Front. Plant Sci. 9. doi: 10.3389/fpls.2018.01250 30271414 PMC6146286

[B58] Traxler C. Gaines T. A. Küpper A. Luemmen P. Dayan F. E. (2023). The nexus between reactive oxygen species and the mechanism of action of herbicides. J. Biol. Chem. 299, 105267. doi: 10.1016/j.jbc.2023.105267 37734554 PMC10591016

[B59] Uffelmann E. Huang Q. Q. Munung N. S. de Vries J. Okada Y. Martin A. R. . (2021). Genome-wide association studies. Nat. Rev. Methods Primers 59:1294–1301. doi: 10.1038/s43586-021-00056-9 41851339

[B60] Vijaya Bhaskar A. V. Vijayakumar I. Torra J. Runge F. Hennessy M. Forristal P. D. (2025). Insights into ALS-inhibiting herbicide resistance in Poa annua in an arable cropping system. Pest Manage. Sci. 81, 8128–8136. doi: 10.1002/ps.70121 40798859 PMC12618908

[B61] Xia F. Quan L. Lou Z. Sun D. Li H. Lv X. (2022). Identification and comprehensive evaluation of resistant weeds using unmanned aerial vehicle-based multispectral imagery. Front. Plant Sci. 13. doi: 10.3389/fpls.2022.938604 35937335 PMC9346607

[B62] Xu P. Wang K. Ju Y. Fu Y. Zhu A. Cao K. . (2025). Herbicide resistance in Leptochloa chinensis (L.) Nees populations from different regions of Jiangsu Province, China: sensitivity differences and underlying mechanisms. Front. Plant Sci. 16. doi: 10.3389/fpls.2025.1535877 39967816 PMC11832499

[B63] Yanniccari M. Palma-Bautista C. Vázquez-García J. G. Gigón R. Mallory-Smith C. A. De Prado R. (2023). Constitutive overexpression of EPSPS by gene duplication is involved in glyphosate resistance in Salsola tragus. Pest Manage. Sci. 79, 1062–1068. doi: 10.1002/ps.7272 36327342

[B64] Zhang Y. Wan J. He L. Lan H. Li L. (2019). Genome-wide association analysis of plant height using the maize f1 population. Plants 8, 432. doi: 10.3390/plants8100432 31640296 PMC6843250

[B65] Zieschank V. Junker R. R. (2023). Digital whole-community phenotyping: tracking morphological and physiological responses of plant communities to environmental changes in the field. Front. Plant Sci. 14. doi: 10.3389/fpls.2023.1141554 37229120 PMC10203609

[B66] Zweifach A. (2024). Samples in many cell-based experiments are matched/paired but taking this into account does not always increase power of statistical tests for differences in means. Mol. Biol. Cell 35:1–8. doi: 10.1091/mbc.E23-05-0159 37910179 PMC10881176

