# Correction: Digital morphological data can generate accurate pre-emergence herbicide dose-response curves in *Chenopodium album* L

**DOI:** 10.3389/fpls.2026.1943599

**Published:** 2026-08-04

**Authors:** Olivia Landau, Jesus Lopez Borges, Raissa Na-ah, Luigi Peracchi, Franco Sanchez-Izurieta

**Affiliations:** 1 United States Department of Agriculture, Agricultural Research Service, Wheat Health, Genetics, and Quality Research Unit, Pullman, WA, United States; 2 Department of Crop and Soil Sciences, Washington State University, Pullman, WA, United States

**Keywords:** atrazine, digital biomass, digital phenotyping, dose-response, fomesafen, morphological data, spectral data

There was a mistake in **Table 3** as published. Two cells of the table were missing content: under the Atrazine section, the Model and GR_50_ estimate cells are blank in the row for CHMW. All other elements of **Table 3** are correct. The corrected **Table 3** appears below.

**Table 3:** Summary of variables in the three-parameter logistic regression and growth reduction (GR_50_) estimates for morphological parameters.

Atrazine				
Parameter^‡^	Model	GR_50_ estimate (SE) ^§^ g ai ha^-1^	Parameter GR_50_:FB GR_50_ ratio (SE)	*P-*value for parameter GR_50_:FB GR_50_ ratio
3DLA	$y=\frac{104.16}{1+\exp\left\{1.67\left[\log\left(x\right)-\log\left(29.57\right)\right]\right\}}$	29.57 (± 8.76)*	0.94 (± 0.40)*	0.8887*
CHA	$y=\frac{94.20}{1+\exp1.76\left[\log\left(x\right)-\log\left(28.36\right)\right]\}}$	28.36 (± 8.58)*	0.90 (± 0.39)*	0.8085*
CHAC	$y=\frac{104.04}{1+\exp\left\{6.81\left[\log\left(x\right)-\log\left(215.37\right)\right]\right\}}$	215.37 (± 112.56)	6.87 (± 4.18)	0.1655
CHAR	$y=\frac{99.05}{1+\exp\left\{2.79\left[\log\left(x\right)-\log\left(126.91\right)\right]\right\}}$	126.91 (± 28.93)	4.05 (± 1.56)	0.0558
CHC	$y=\frac{100.34}{1+\exp\left\{0.67\left[\log\left(x\right)-\log\left(74.18\right)\right]\right\}}$	74.18 (± 43.61)	2.37 (± 1.57)	0.3881
CHMW	$y=\frac{104.35}{1+\exp\left\{0.44\left[\log\left(x\right)-\log\left(102.52\right)\right]\right\}}$	102.52 (± 112.87)	3.27 (± 3.74)	0.5465
CLPD	$y=\frac{98.14}{1+\exp\left\{1.56\left[\log\left(x\right)-\log\left(77.17\right)\right]\right\}}$	77.17 (± 22.46)	2.46 (± 1.05)	0.1687
DB	$y=\frac{106.48}{1+\exp\left\{2.39\left[\log\left(x\right)-\log\left(27.05\right)\right]\right\}}$	27.05 (± 5.58)*	0.86 (± 0.32)*	0.6713*
FB	$y=\frac{95.67}{1+\exp\left\{1.71\left[\log\left(x\right)-\log\left(31.36\right)\right]\right\}}$	31.36 (± 9.75)	NA	NA
PH Max	$y=\frac{97.44}{1+\exp\left\{1.08\left[\log\left(x\right)-\log\left(81.65\right)\right]\right\}}$	81.65 (± 31.44)	2.60 (± 0.19)	0.0020
PH Mean	$y=\frac{97.65}{1+\exp\left\{1.05\left[\log\left(x\right)-\log\left(82.49\right)\right]\right\}}$	82.49 (± 32.24)	2.63 (± 0.19)	0.0019
PH True	$y=\frac{97.60}{1+\exp\left\{1.07\left[\log\left(x\right)-\log\left(63.88\right)\right]\right\}}$	63.88 (± 24.57)	2.04 (± 0.24)	0.0404
PLA	$y=\frac{100.07}{1+\exp\left\{1.84\left[\log\left(x\right)-\log\left(31.74\right)\right]\right\}}$	31.74 (± 9.04)*	1.01 (± 0.42)*	0.9770*
VVT	$y=\frac{99.98}{1+\exp\left\{1.73\left[\log\left(x\right)-\log\left(31.96\right)\right]\right\}}$	31.96 (± 9.37)*	1.02 (± 0.42)*	0.9643*
Fomesafen				
Parameter	Model	GR_50_ estimate (SE) g ai ha^-1^	Parameter GR_50_:FB GR_50_ ratio (SE)	*P-*value for parameter GR_50_:FB GR_50_ ratio
3DLA	$y=\frac{100.82}{1+\exp\left\{2.54\left[\log\left(x\right)-\log\left(9.55\right)\right]\right\}}$	9.55 (± 2.16)*	1.00 (± 0.32)*	0.9977*
CHA	$y=\frac{104.88}{1+\exp\left\{3.03\left[\log\left(x\right)-\log\left(10.65\right)\right]\right\}}$	10.65 (± 2.21)*	1.11 (± 0.34)*	0.7423*
CHAC	$y=\frac{90.00}{1+\exp\left\{3.67\left[\log\left(x\right)-\log\left(87.09\right)\right]\right\}}$	87.09 (± 24.42)	9.11 (± 3.30)	0.0169
CHAR	$y=\frac{98.80}{1+\exp\left\{3.29\left[\log\left(x\right)-\log\left(99.26\right)\right]\right\}}$	99.26 (± 23.96)	10.38 (± 3.46)	0.0086
CHC	$y=\frac{116.29}{1+\exp\left\{0.67\left[\log\left(x\right)-\log\left(22.32\right)\right]\right\}}$	22.32 (± 10.79)	2.33 (± 1.25)	0.2895
CHMW	$y=\frac{118.60}{1+\exp\left\{0.53\left[\log\left(x\right)-\log\left(26.40\right)\right]\right\}}$	26.40 (± 17.78)	2.76 (± 1.96)	0.3732
CLPD	$y=\frac{112.41}{1+\exp\left\{1.17\left[\log\left(x\right)-\log\left(19.07\right)\right]\right\}}$	19.07 (± 5.69)	1.99 (± 0.75)	0.1903
DB	$y=\frac{106.90}{1+\exp\left\{3.51\left[\log\left(x\right)-\log\left(7.93\right)\right]\right\}}$	7.93 (± 1.83)*	0.83 (± 0.26)*	0.5291*
FB	$y=\frac{100.69}{1+\exp\left\{3.10\left[\log\left(x\right)-\log\left(9.56\right)\right]\right\}}$	9.56 (± 2.19)	NA	NA
PH Max	$y=\frac{116.35}{1+\exp\left\{0.77\left[\log\left(x\right)-\log\left(35.34\right)\right]\right\}}$	35.34 (± 14.85)	3.70 (± 0.13)	4.9463 x 10^-7^
PH Mean	$y=\frac{116.88}{1+\exp\left\{0.76\left[\log\left(x\right)-\log\left(38.21\right)\right]\right\}}$	38.21 (± 16.26)	4.00 (± 0.12)	5.6220 x 10^-8^
PH True	$y=\frac{113.54}{1+\exp\left\{1.52\left[\log\left(x\right)-\log\left(18.05\right)\right]\right\}}$	18.05 (± 4.57)	1.89 (± 0.18)	0.0117
PLA	$y=\frac{101.20}{1+\exp\left\{2.55\left[\log\left(x\right)-\log\left(9.64\right)\right]\right\}}$	9.64 (± 2.15)*	1.01 (± 0.32)*	0.9785*
SA Mean	$y=\frac{209.50}{1+\exp\left\{-0.04\left[\log\left(x\right)-\log\left(55.59\right)\right]\right\}}$	55.59 (± 1521.43)	5.81 (± 0.42)	0.8610
VVT	$y=\frac{102.77}{1+\exp\left\{2.44\left[\log\left(x\right)-\log\left(9.75\right)\right]\right\}}$	9.75 (± 2.14)*	1.02 (± 0.31)*	0.9516*

^†^For each experiment, parameters that generated dose-response curves and GR50 estimates similar to FB data are highlighted with ‘*’. ^‡^3DLA, 3D leaf area; CHA, convex hull area; CHAC, convex hull area coverage; CHAR, convex hull aspect ratio; CHC, convex hull circumference; CHMW, convex hull max width; CLPD, canopy light penetration depth; DB, digital biomass; FB, fresh biomass; PH Max, plant height max; PH Mean, plant height mean; PH True, plant height true; PLA, projected leaf area; SA Mean, surface area mean; VVT, voxel volume total. ^§^SE, standard error of the mean.

The original version of this article has been updated.

