# Supplementary figures and images for: Digital morphological data can generate accurate pre-emergence herbicide dose-response curves in Chenopodium album L
Source: Front Plant Sci. 2026 Apr 21;17:1779398. doi: 10.3389/fpls.2026.1779398 (PMC13139323; doi:10.3389/fpls.2026.1779398)

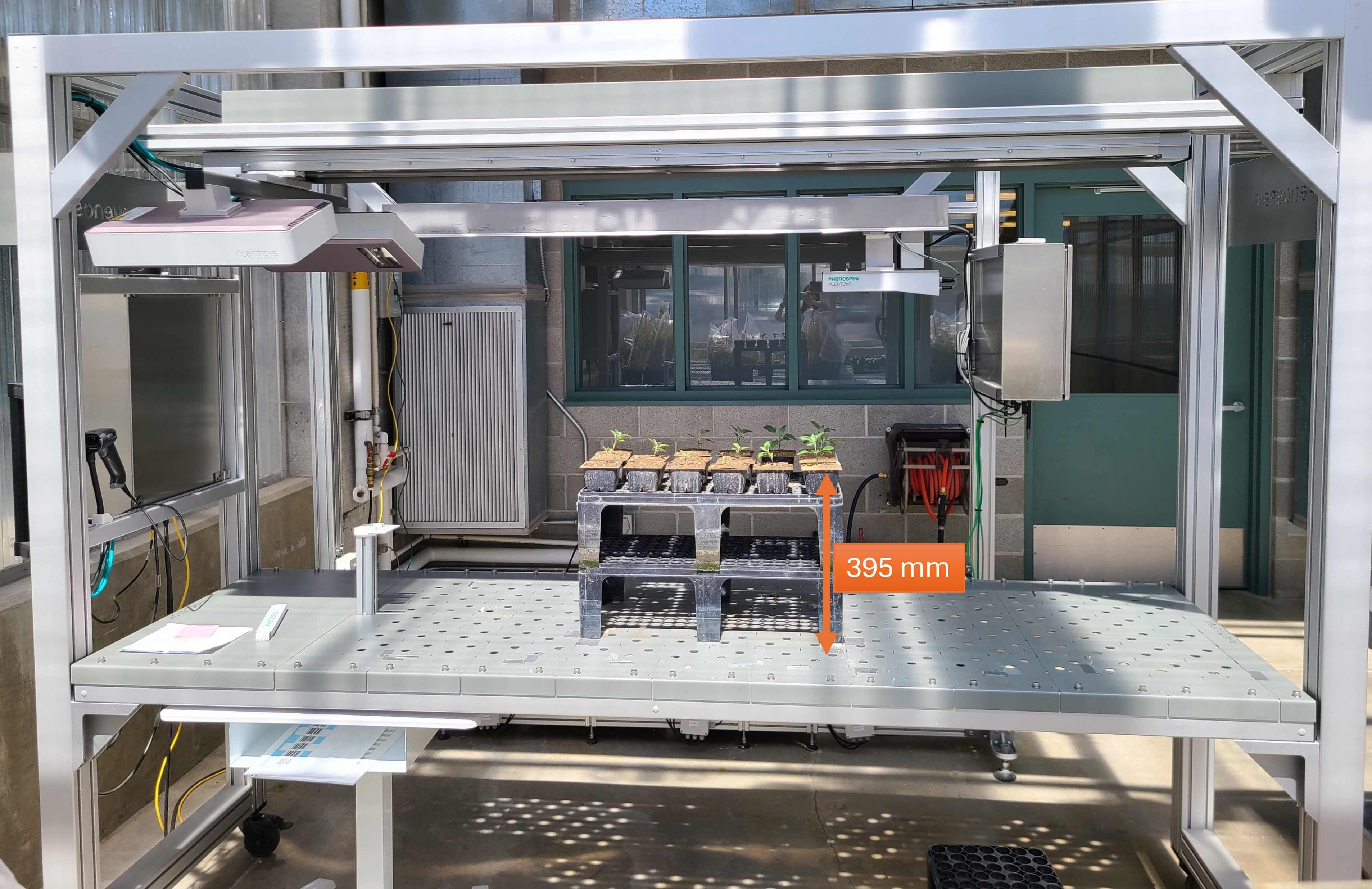

Supplement: Supplementary Figure S1 — The Phenospex TraitFinder utilized for this research. Pots were arranged in a 3x6 pattern and elevated to a height of 395 mm. [file Image1.jpeg]

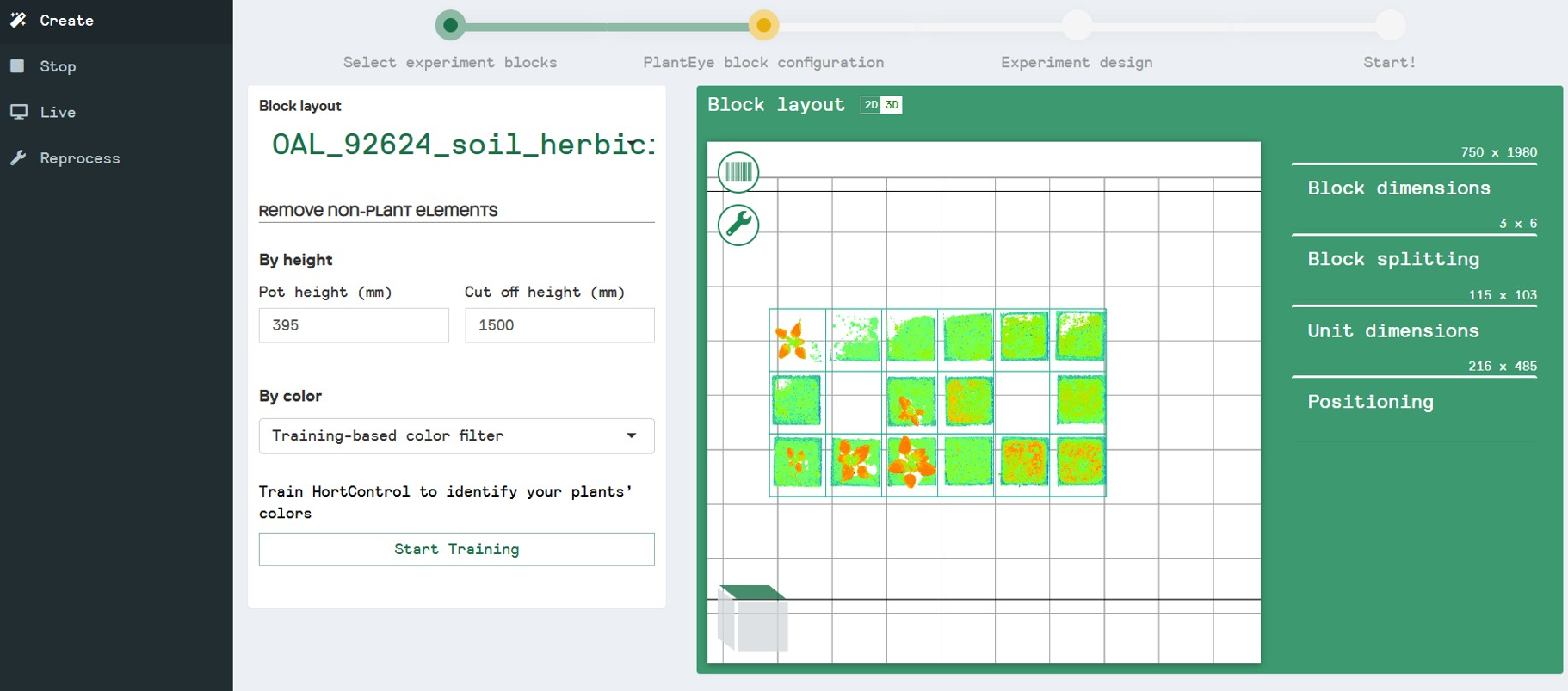

Supplement: Supplementary Figure S2 — An example of HortControl block configuration. Pots were arranged in a 3x6 grid, with each cell of the grid containing one pot from which data was collected. A color filter for normalized difference vegetation index is currently implemented to make the plants and pots more visible during block configuration. The color filter was not utilized for data collection. [file Image2.jpeg]

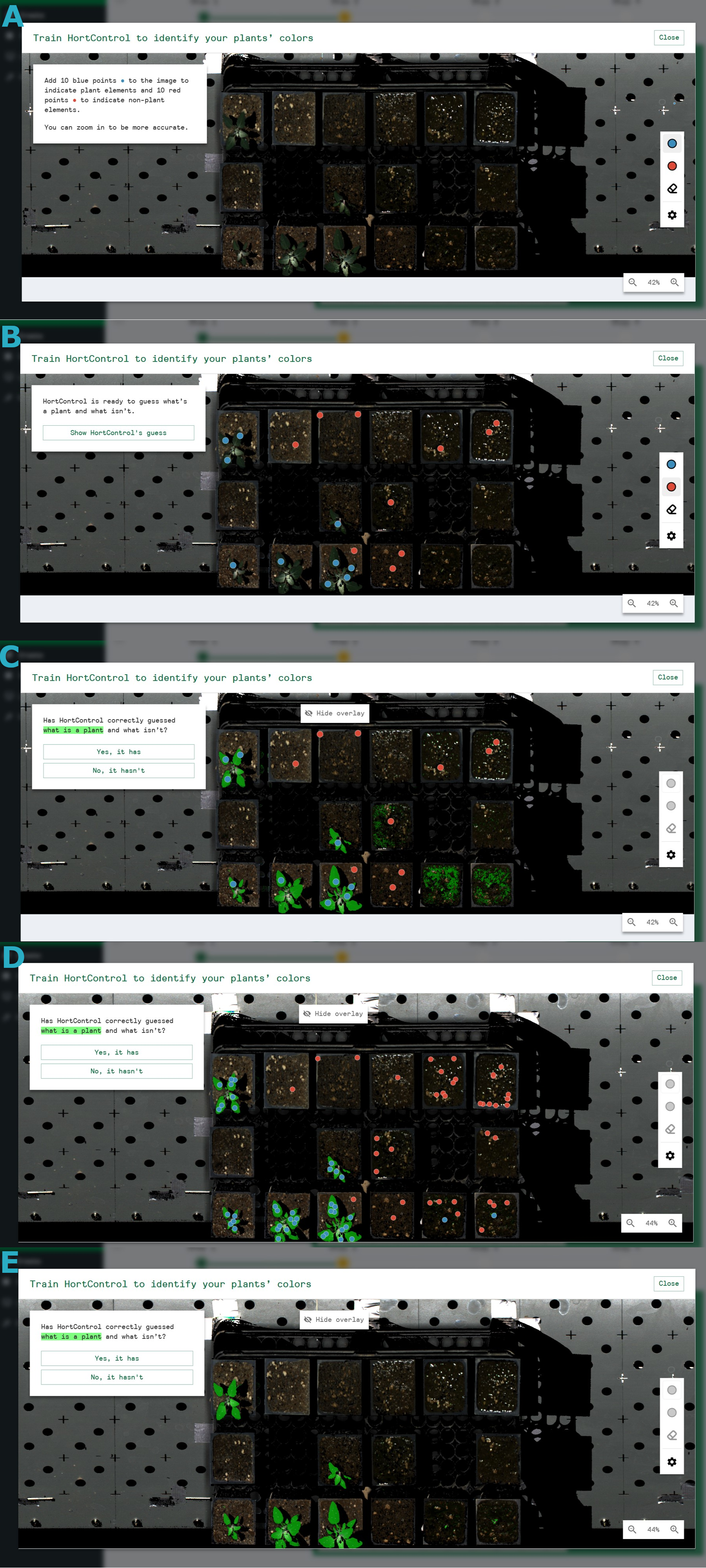

Supplement: Supplementary Figure S3 — An example setup for the training-based color filter using the same plants from Supplementary Figure 2. Part of the randomized complete block design involved arranging pots in a 3x6 patten that included two blank spots to serve as a negative control for PlantEye F600 3D scanners. Figure (A) depicts the red-green-blue image acquired by the PlantEye F600 3D scanners. Ten blue dots are assigned to plant material and ten red dots are assigned to non-plant material (e.g. soil, pot edges, perlite, etc.) to train HortControl, which is depicted in Figure (b). Figure (C) shows HortControl’s initial guess based on the input from Figure (B). Figure (D) displays additional dots being added to improve HortControl training. Figure (E) indicates successful differentiation between plant and non-plant material by HortControl. Note that HortControl is detecting two small seedlings located in third row of the 5th and 6th columns, which were not easily apparent in Supplementary Figure 2. [file Image3.jpeg]
